# Characterization of CD4^+^ T Cell Subsets in Patients with Abdominal Aortic Aneurysms

**DOI:** 10.1155/2018/6967310

**Published:** 2018-12-27

**Authors:** Fábio Haach Téo, Rômulo Tadeu Dias de Oliveira, Liana Villarejos, Ronei Luciano Mamoni, Albina Altemani, Fabio Husemann Menezes, Maria Heloisa Souza Lima Blotta

**Affiliations:** ^1^Department of Clinical Pathology, Faculty of Medical Sciences, State University of Campinas (UNICAMP), Campinas, São Paulo 13083-887, Brazil; ^2^Faculty of Medicine of Jundiai, Jundiai, São Paulo 13202-550, Brazil; ^3^Department of Pathology, Faculty of Medical Sciences, State University of Campinas (UNICAMP), Campinas, São Paulo 13083-887, Brazil; ^4^Department of Surgery, Faculty of Medical Sciences, State University of Campinas (UNICAMP), Campinas, São Paulo 13083-887, Brazil

## Abstract

**Background:**

The mediators produced by CD4^+^ T lymphocytes are involved in the pathogenesis of aneurysmal lesions in abdominal aortic aneurysm (AAA) patients. The aim of this study was to identify and characterize the CD4^+^ T cell subsets involved in human AAA.

**Methods:**

The CD4^+^ T cell subsets in 30 human aneurysmal lesions were determined using flow cytometry (FC) and immunohistochemistry (IHC). The peripheral blood mononuclear cells (PBMCs) from patients with AAA were also analyzed by FC and compared with control subjects.

**Results:**

Human aneurysmal lesions contained IFN-*γ*, IL-12p35, IL-4, IL-23p19, IL-17R, and IL-22 positive cells. PBMCs from AAA patients had higher expression levels of IFN-*γ*, TNF-*α*, IL-4, and IL-22 when compared to controls.

**Conclusions:**

Our results show the presence of T_H_1, T_H_2, T_H_17, and T_H_22 subsets in aneurysmal lesions of AAA patients and suggest that these cells may be mainly activated in situ, where they can induce tissue degradation and contribute to the pathogenesis of AAA.

## 1. Introduction

Abdominal aortic aneurysm (AAA) is a degenerative disorder characterized by a progressive weakening of the abdominal aortic wall, resulting in an increase in vessel diameter of at least 50% [1]. Although the morbidity of the disease varies worldwide, it may affect 9% of males who are 65 years old, and mortality may reach 90% in complicated cases, caused by internal hemorrhage due to rupture of the damaged abdominal aorta [2].

The deleterious modifications that occur during the development of AAA mainly affect the tunica media, where degradation of extracellular matrix components and apoptosis of vascular smooth muscle cells take place [3]. The triggers of these pathological processes are not fully understood, but epidemiological studies have associated factors such as smoking, high blood pressure, and the male gender with aortic wall injury [4]. The recruitment of leukocytes into the aortic media, mediated by chemokines and elastin degradation products, appears to be an early and pivotal event in the pathogenesis of AAA [5]. Indeed, the presence of extensive inflammatory infiltrates is the histological hallmark of aneurysmal lesions in AAA [6], and increased levels of inflammatory cytokines such as TNF-*α*, IL-6, and IL-1*β* have been reported in the sera or plasmas of AAA patients [7]. Genome-wide analyses of AAA tissue provide evidence of upregulation of genes involved in leukocyte migration, activation of immune response, activation of T cell, and regulation of lymphocyte activation whereas the downregulated genes were enriched in the following categories: cytoskeleton organization, muscle cell development, organ morphogenesis, and cell junction assembly [8]. In addition, patients with AAA and aortic occlusive disease overexpress the inflammasome NLRP3 mRNA indicating a common inflammatory etiology of both diseases [9]. Expression of genes implicated in inflammation and matrix degradation such as COX2, MMP-2, and MMP-9 was also described as elevated in aneurysm tissue [10, 11]. These findings suggest that immune-inflammatory mechanisms may participate in the development of AAA.

Several immune cell types have been identified in AAA lesions, including macrophages [12] and lymphocytes [6]. Most of the lymphocytes are identified as CD4^+^ T cells. These cells can be divided into different subtypes according to the production of their signature cytokines and the expression of specific transcription factors. T_H_1 cell differentiation is dependent on the presence of IL-12 and the expression of the transcription factor T-bet, while T_H_2 cell generation occurs when there is IL-4 in the environment, leading to the expression of GATA-3 and c-MAF transcription factors [13]. The signature cytokines of T_H_1 lymphocytes are TNF-*α* and IFN-*γ*, while that of T_H_2 cells is IL-4. Several reports demonstrate that IFN-*γ* and IL-4 might contribute to the pathogenesis of AAA by inducing macrophages and vascular smooth muscle cells to produce different types of metalloproteinases (MMPs) and cathepsins. These enzymes lead to aortic wall deterioration mainly by collagen and elastin degradations [14, 15]. Although researchers suggest the presence of T_H_1 and T_H_2 in human lesions [16], the role of these cells in the pathogenesis of AAA is not yet fully understood.

CD4^+^ T cells may also differentiate into T_H_17 and T_H_22 subsets [17, 18]. The expression of transcription factor RORC in the presence of TGF-*β* and IL-6 leads to T_H_17 generation [19], while the IL-6- and TNF-*α*-dependent expressions of AHR characterize the T_H_22 subset [18]. The signature cytokines of T_H_17 cells are IL-17 and IL-23 [20], while the signature cytokine of T_H_22 cells is IL-22 [18]. Several reports show that IL-17 is capable of inducing the generation of several inflammatory mediators such as IL-1, IL-6, TNF-*α*, CCL2, CCL7, CCL20, CXCL1, CXCL8, and MMPs by endothelial cells, smooth muscle cells, and macrophage [21, 22]. Similarly, IL-22 has an inflammatory potential to induce the production of MMPs, CCL7, CXCL1, CXCL3, CXCL9, CXCL8, and IL-6 by fibroblasts [23]. These mediators, induced by IL-17 and IL-22, may contribute to the degradation of tunica media of the abdominal aorta.

Recent epidemiological studies have reported the association between diseases which are known to be mediated by T_H_ cell subsets and AAA. A Danish population-based nationwide case-control study showed that asthma, a T_H_2-driven pathological condition, increases risk of AAA development and rupture [24]. Moreover, a nationwide cohort study also comprising the Danish population reported psoriasis was associated with increased risk of AAA, and the risk appears to increase with the severity of psoriasis [25]. IL-17 is a key cytokine in psoriasis pathogenesis, and inhibition of T_H_17 cells is a therapy strategy [26]. T_H_22 cells and their signature cytokine also have been associated with psoriasis [27].

It is evident that different subsets of T_H_ cells participate in the development of characteristic pathological alterations observed in human AAA, and recent studies are shedding light in the mechanisms by which the different T_H_ cell subtypes may modulate MMP production, contributing to aorta wall weakening [12, 28]. According to microenvironment cytokines e survival factors, most of them produced by T_H_ cells, macrophages are either “classically activated” (M1) by TNF-*α* and IFN-*γ* or “alternatively activated” (M2) by IL-4, IL-13, and IL-10. M1 and M2 macrophages differ in their MMPs and TIMP production profiles [28]. T_H_ cells, mainly T_H_2 cells, may also contribute to AAA by activating B cells and leading to antibody production [29]. B cells, along with T cells, are the most frequent lymphocyte subsets in AAA cases [30]. Nevertheless, despite the increasing available data, the role of the different T_H_ cells in AAA is not fully understood. Thus, in this study, we aimed to identify and characterize the different CD4^+^ T cell subsets in patients with AAA.

## 2. Materials and Methods

### 2.1. Patients and Controls

Thirty patients who were submitted to elective open repair of an AAA, attending the Clinical Hospital, State University of Campinas (UNICAMP), Campinas, a tertiary referral hospital, were enrolled in this study.

The control group was composed of individuals with the same comorbidities present in the patients with AAA but without documented disease. Exclusion criteria included decompensated congestive heart failure, clinically significant valvular heart disease, known or suspected thrombotic disorders, malignancies, inflammatory or autoimmune disorders, a history of myocardial infarction within the preceding month, or renal or pulmonary failure.

Informed consent was obtained from all participants, and the study protocol conformed to the ethical guidelines of the 1975 Declaration of Helsinki, reflected in the approval by the Ethical Committee of UNICAMP.

### 2.2. Processing AAA Lesion Samples

AAA lesion samples were collected during open surgical repair and were subjected to different procedures. Samples for immunohistochemistry (IHC) were fixed in 4% formaldehyde for 24 h, embedded in paraffin, and sectioned into 4 *μ*m slices. The samples used in cellular infiltrate phenotyping by flow cytometry were placed in RPMI 1640 medium until the samples arrived at the laboratory. The samples were then enzymatically digested overnight with liberase (2 mg/ml; Blendzymes—Roche, Sigma-Aldrich, St Louis., MO, USA). The samples were further disintegrated using Medimachine (BD Biosciences, San Jose, CA, USA). The resulting cellular suspension was expanded *in vitro* for 10 days in RPMI 1640 medium (Invitrogen Co., Grand Island, NY, USA) supplemented with glutamine (2 mM; Sigma, St. Louis, USA), gentamycin (5 *μ*g/ml; Sigma-Aldrich), 10% heat-inactivated human AB serum, and IL-2 (10 ng/ml, R&D Systems, Minneapolis, MN, USA) at 37°C in 5% CO_2_. After expansion, the mononuclear cells (MCs) were separated using a Ficoll-Hypaque density gradient (Amersham Biosciences, Uppsala, Sweden).

### 2.3. Immunohistochemistry

Sections of AAA lesions were immersed in 5% Trilogy solution (Sigma-Aldrich) and placed in a steamer for 15 min in order to deparaffinize, hydrate, and recover the antigens. Endogenous peroxidase was blocked with H_2_O_2_ (10x volume), and each section was incubated with CD3, CD8 (Dako, Santa Clara, CA, USA), IL-4, IL-23 (BioLegend, San Diego, CA, USA), IL-17 (Santa Cruz, Dallas, TX, USA), or IL-22 antibodies (Abcam, Cambridge, UK). NovoLink Polymer Detection System (Novocastra, Newcastle, England) was used according to the manufacturer's instructions to detect any tissue-bound antibody. Color development was carried out with a 3,3′-diaminobenzidine (DAB) solution, and the sections were counterstained with hematoxylin.

### 2.4. Blood Sampling

Blood samples from patients, one day before open surgical repair, and from control individuals were collected into heparin tubes. Once collected, the blood samples were centrifuged in Ficoll-Hypaque (GE Healthcare, Piscataway, NJ, USA), and the peripheral blood mononuclear cells (PBMCs) were obtained. Plasma samples were stored at −80°C for high sensitivity C-reactive protein (hs-CRP) quantification.

### 2.5. *In Vitro* Stimulation of Mononuclear Cells

For detection using flow cytometry, mononuclear cells (MCs) from peripheral blood and from AAA lesions were stimulated *in vitro* by the signature cytokines produced by each T helper subset. The cells were cultured at a concentration of 2 × 10^6^ cells per well in 24-well plates (Amersham Biosciences, Uppsala, Sweden) in the presence of RPMI 1640 medium supplemented with glutamine (2 mM), gentamycin (5 *μ*g/ml), and 10% heat-inactivated human AB plasma. PBMCs were stimulated with anti-CD3, anti-CD28, and anti-CD2 antibody-coated beads (Miltenyi) for 48 h at 37°C in 5% CO_2_. In the final 6 h, phorbol myristate acetate (PMA, 50 ng; Sigma-Aldrich), calcium ionophore (250 ng; Sigma-Aldrich), and brefeldin A (1 *μ*g; Sigma-Aldrich) were added to the medium. MCs from AAA lesions were initially incubated with anti-CD3, anti-CD28, and anti-CD2 antibody-coated beads for 10 days at 37°C in 5% CO_2_ in order to expand T lymphocytes. During the final 6 h of incubation, the cells were harvested, counted (2 × 10^6^ cells/well), and stimulated, as previously described for PBMCs.

### 2.6. Flow Cytometry

After stimulation, *in vitro* PBMCs and MCs from AAA lesions were harvested and analyzed by flow cytometry for T cell population phenotypes. Each immunofluorescence reaction was carried out using 3 × 10^5^ cells diluted in PBS. Initially, the cells were incubated with specific antibodies for surface markers: CD3 (FITC, PE (Caltag), and PerCP (BioLegend)), CD8 (APC (BioLegend) and FITC (BD Biosciences)), CCR5 (FITC (BD Biosciences)), CCR6 (PE (BioLegend)), CXCR3 (PE (BD Biosciences)), and CCR4 (PerCP/Cy5.5 (BioLegend)). Next, the cells were fixed in 2% formaldehyde, permeabilized with saponin (0.5% in PBS; Sigma-Aldrich), and subjected to intracellular staining for IFN-*γ* (FITC—BD Biosciences), TNF-*α* (PE—BD Biosciences), IL-4 (APC—BioLegend), IL-17 (PE—eBioscience), and IL-22 (eFluor 660—eBioscience, San Diego, CA, USA). The cells were acquired on a FACSCalibur flow cytometer (BD Biosciences, San Jose, CA, USA). The analysis was carried out using FCS Express software (De Novo Software, Glendale, CA, USA).

### 2.7. Statistical Analysis

**Figure 1:**
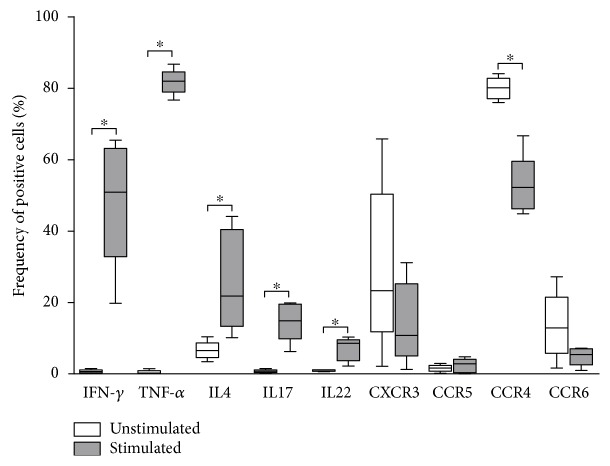
Frequency (%) of CD4^+^ lymphocytes (T_H_ cells) positive for the signature cytokines and homing molecules of the different T_H_ subsets (T_H_1, T_H_2, T_H_17, and T_H_22) in mononuclear cell suspensions of abdominal aortic aneurysm lesions (*N* = 5). The mononuclear cell suspensions were expanded *in vitro* for 15 days and left without stimulation (white) or were stimulated by PMA plus calcium ionophore for the last 6 h of incubation (grey). ^∗^ *P* < 0.05; Wilcoxon matched pairs test.

**Figure 2 fig2:**
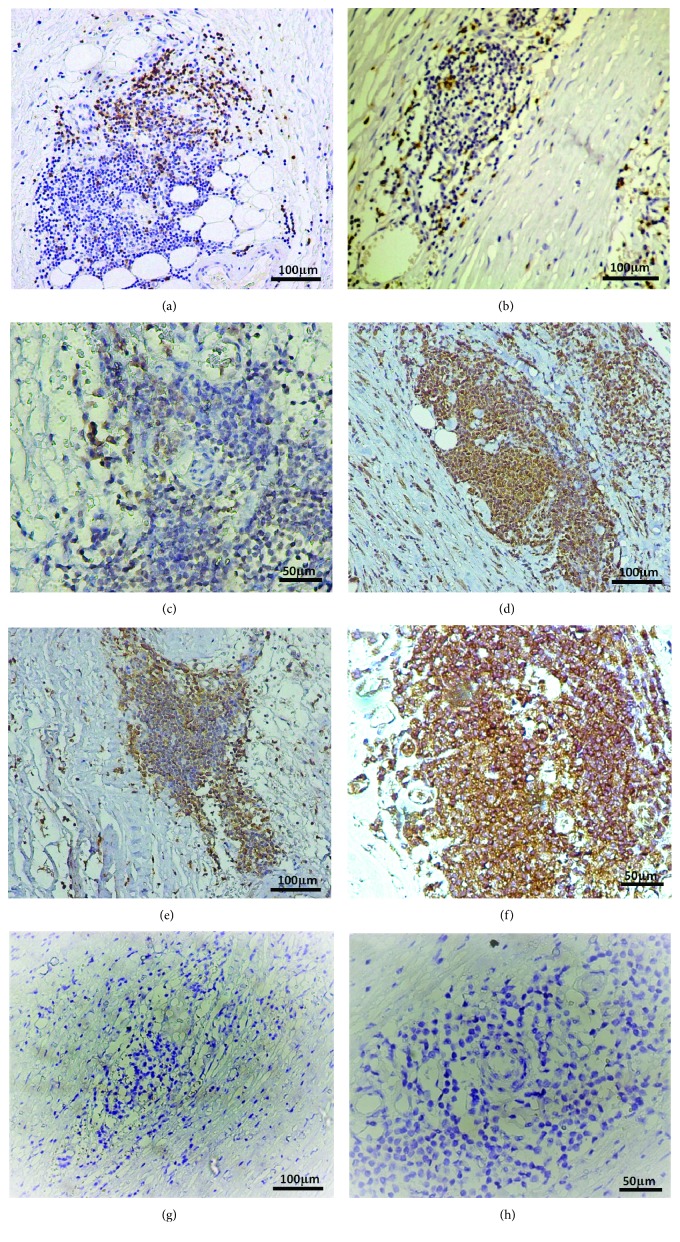
Paraffin-embedded tissue sections of AAA lesions (*N* = 16) were immunohistochemically labeled (brown) for CD3^+^ cells (a), CD8^+^ cells (b), IL-4^+^ cells (c), IL-17^+^ cells (d), IL-23^+^ cells (e), and IL-22^+^ cells (f) in follicle-like structures located in the adventitia and media of abdominal aortic aneurysm lesions. (g) and (h) show negative controls (without the primary antibody) at different magnifications.

**Figure 3:**
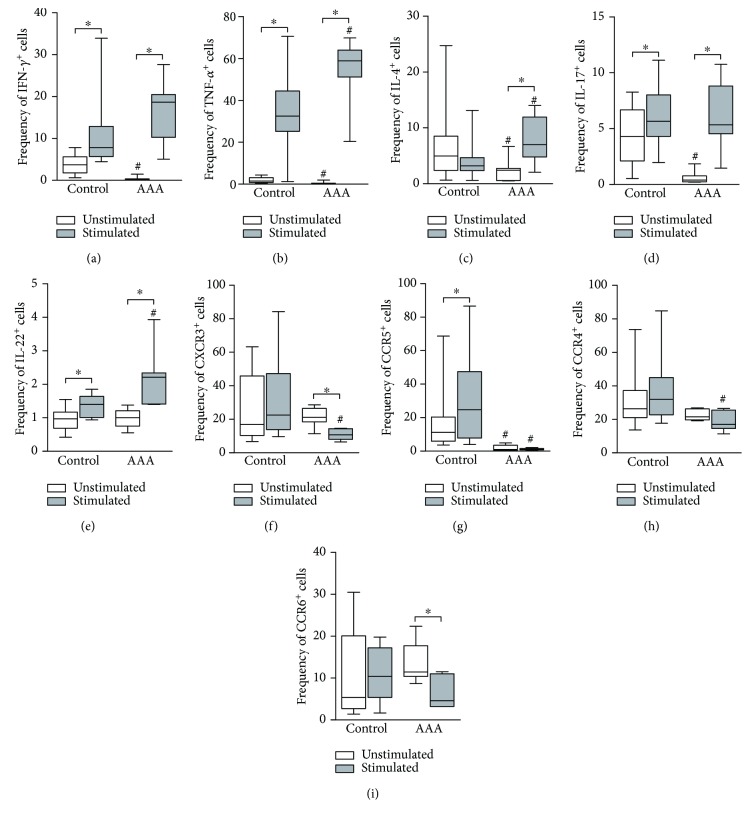
Frequency (%) of CD4^+^ (T_H_) cells positive for the signature cytokines (a–e) and *homing* molecules (f–i) of the different T_H_ subsets (T_H_1, T_H_2, T_H_17, and T_H_22) in peripheral blood mononuclear cell samples from control subjects (*N* = 11) and patients with abdominal aortic aneurysm (*N* = 12). The analyzed samples were cultured for 48 h in the absence of stimulus (white) or in the presence of anti-CD3, anti-CD28, and anti-CD2 antibody-coated beads (grey), with the addition of PMA plus calcium ionophore in the last 6 h of incubation. *P* < 0.05; ^∗^comparison between stimulated and unstimulated cells (Wilcoxon matched pairs test); ^#^comparison between control cells and AAA peripheral cells (Mann-Whitney test).

The statistical analysis was performed using GraphPad Prism software, 5.0 (La Jolla, CA, USA). All measurements (Figures 1 [Fig fig2]–3) are represented as the median and interquartile range, and *P* values < 0.05 are considered statistically significant. Two group comparisons (AAA vs. control and AAA vs. peripheral blood) of the quantitative data were performed using the nonparametric Mann-Whitney rank sum test. Parameters before and after mononuclear cell stimulation in culture were compared using the nonparametric Wilcoxon matched pairs test.

## 3. Results

### 3.1. Characterization of the Study Groups

Demographic and clinical features of the patients with AAA enrolled in this study are summarized in Table 1. The experimental group was composed mainly of men (the male/female ratio was 28/2), with an average age of 65.4 years (range 60.3–70.5 years). Regarding the stage of the disease, the mean diameter of the abdominal aortae was 6.5 cm (range 4.9–8.1 cm), and the thickness of the intraluminal thrombi was 2.8 cm (range 0.8–4.8 cm). The majority of the patients had a current or prior smoking habit associated with hypertension. Other comorbidities found in the experimental group were dyslipidemia, diabetes, and obesity. The therapeutic regimens of the patients indicated a frequent presence of AAA-associated cardiovascular diseases, atherosclerosis being the most common pathophysiologic process. However, biochemical data suggest these comorbidities were attenuated, except for elevated levels of CRP, a marker of inflammation.

The control group was younger and showed an equal distribution between male and female sexes compared to patients with AAA. It also presented a higher frequency of obese individuals in addition to a tendency of diabetes and dyslipidemia, which may have influenced the frequent use of statins. In fact, different from the majority, the patients with AAA submitted to elective open repair; the control group was mostly composed of patients with metabolic syndrome, with hypertension, diabetes, dyslipidemia, and obesity equally recurrent in the group. Also, in the control group, cardiovascular disease was less common.

### 3.2. AAA Lesion Analysis

#### 3.2.1. Phenotyping

The phenotyping of unstimulated MCs from AAA lesions showed a very low percentage of T_H_ cells producing their signature cytokines (Figure 1). Of the MCs, less than 1% were positive for IFN-*γ*, TNF-*α*, and IL-17. IL-4 was present in 6.6% of the CD4^+^ T cells, while IL-22 was positive in 1.1% of the population. After stimulation with PMA and calcium ionophore, we detected a high percentage of cells producing either IFN-*γ* (50.9%) or TNF-*α* (82%). Most of the cells were IFN-*γ* ^+^TNF-*α* ^+^, and the T_H_1 signature cytokines were the most prevalent among the T_H_ cells from AAA. The main T_H_2 cytokine, IL-4, was present in 21.8% of the analyzed population after *in vitro* stimulation. We also observed that 14% of the stimulated CD4^+^ T cells were producing only IL-17, while IL-22 single-positive cells represented 8.5% of the population. Indeed, we did not find a significant number of IL-17 and IL-22 double-positive cells.

Regarding chemokine receptors expressed in T_H_2, T_H_17, and T_H_22, most of unstimulated T_H_ cells from AAA lesions were CCR4^+^ (80.14%). Only a few cells were CCR4^+^CCR6^+^ cells, and 12.85% of the analyzed cells were only CCR6^+^. The T_H_1 specific receptor CXCR3 was present in 23.33% of CD4^+^ T lymphocytes, and only 1.7% of the population was CCR5^+^. After *in vitro* stimulation, we observed a similar pattern of expression of chemokine receptors, with CCR4 and CXCR3 being the most prevalent. We detected a decrease in the frequency of positive cells for CCR4 and CCR6 after *in vitro* stimulation. The percentage of CXCR3^+^ cells also decreased, but the difference did not reach statistical significance. The frequency of CCR5^+^ cells was very low and did not change after stimulation (Figure 1).

Taken together, our first set of data shows that, although the signature cytokines for T_H_1, T_H_2, T_H_17, and T_H_22 were almost absent in unstimulated cells, the intralesional lymphocytes can produce large quantities of the cytokines following stimulation.

#### 3.2.2. Immunohistochemistry

The T_H_ cell subsets within AAA lesions in tissue sections were analyzed by IHC for specific markers. We detected CD3^+^ T cells mainly in follicle-like structures located in the adventitia and media of the aortas (Figure 2(a)). Only a few CD3^+^ cells were found throughout the extracellular matrix components.

We further examined the T lymphocytes within AAA lesions to determine whether most of these cells had a helper or cytotoxic function. CD8^+^ cells were detected inside inflammatory infiltrates, but CD8^−^ cells were higher in number; these cells belonged to the helper population (Figure 2(b)). Indeed, we further identified either IL-4^+^, IL-17^+^, IL-23^+^, or IL-22^+^ regions in the infiltrates. We found a greater number of positive cells in follicle-like structures, corresponding to the localization of CD3^+^ T cells (Figures 2(c)–2(f)). Importantly, we found IL-17^+^ T cell regions, as well as IL-23^+^ regions.

### 3.3. Peripheral Blood Cell Analysis

We next analyzed the peripheral blood of patients with AAA and control subjects regarding the expression of T_H_ signature cytokines. The cells were evaluated before and after *in vitro* stimulation (Figures 3(a)–3(e)). Only few cells from the patients produced the analyzed inflammatory cytokines prior to stimulation. However, *in vitro* activation with the combination of anti-CD3 and anti-CD28 antibodies induced IFN-*γ*, TNF-*α*, IL-4, IL-17, and IL-22 production. As reported for the cellular infiltrate of AAA lesions, the most prevalent cytokines in stimulated peripheral blood CD4^+^ T cells were TNF-*α* and IFN-*γ* (58.92% and 18.65%, respectively), followed by IL-4 (6.99%), IL-17 (5.35%), and IL-22 (2.21%). The majority of TNF-*α* ^+^ cells were also IFN-*γ* ^+^, while the IL-4^+^, IL-17^+^, and IL-22^+^ cells were mainly single positive. In the control group, we found the same pattern of cytokine production for IFN-*γ*, TNF-*α*, and IL-22 and IL17. However, the IL-4^+^ cells diminished after stimulation but failed to reach significant difference.

Regarding chemokine receptors (Figures 3(f)–3(i)), CCR4 and CXCR3 were the most prevalent not only in unstimulated but also in *in vitro* activated T_H_ cells, followed by CCR6 and CCR5. Interestingly, almost all cells were single positive, and the frequency of CXCR3 and CCR6 within the analyzed population decreased after stimulation, while CCR4 and CCR5 frequency did not change.

Comparable to the experimental group, CCR4 and CXCR3 were the most frequent receptors in unstimulated T_H_ cells from the peripheral blood of control participants. CCR5 was present in a significant percentage of the cells (11.2%), and CCR6 was expressed in only 5.35%. In fact, most of CXCR3^+^ cells were also CCR5^+^ in this group, whereas CCR4^+^ and CCR6^+^ cells were mainly single positives. Unlike in AAA patients, there was an increase in the frequency of CCR5^+^ cells in the control subjects, while the frequency of other receptors did not change after PBMC stimulation.

By comparative analysis of the peripheral T_H_ cell population from AAA patients and controls, we detected a higher frequency of unstimulated IFN-*γ* ^+^ cells in the latter. The result was similar for TNF-*α*, IL-17, and IL-4 (Figures 3(a)–3(e)). However, the frequencies were still very low; thus, this probably does not reflect a biological process. In fact, the main differences between the unstimulated peripheral T_H_ lymphocytes from the analyzed groups were in the expression of chemokine receptors. CCR6^+^ and CXCR3^+^ cells were less prevalent among cells from control participants compared to those from AAA patients, while the frequency of both CCR4 and CCR5 was higher (and statistically significant for CCR5) in the control participants.

When we compared stimulated peripheral T_H_ cells from AAA patients and those from control individuals, there was a higher percentage of cells producing their signature cytokines in the peripheral blood of patients. In fact, AAA patients had increased frequencies of IFN-*γ* ^+^TNF-*α* ^+^ T_H_1, IL-4^+^ T_H_2, and IL-22^+^ T_H_22 cells in the periphery. The only exception was IL-17^+^ T_H_17 lymphocytes, which were equally frequent in peripheral blood from patients and from control subjects. We also detected a decreased percentage of peripheral CXCR3^+^, CCR5^+^, and CCR4^+^ T_H_ cells in AAA patients.

**Figure 4:**
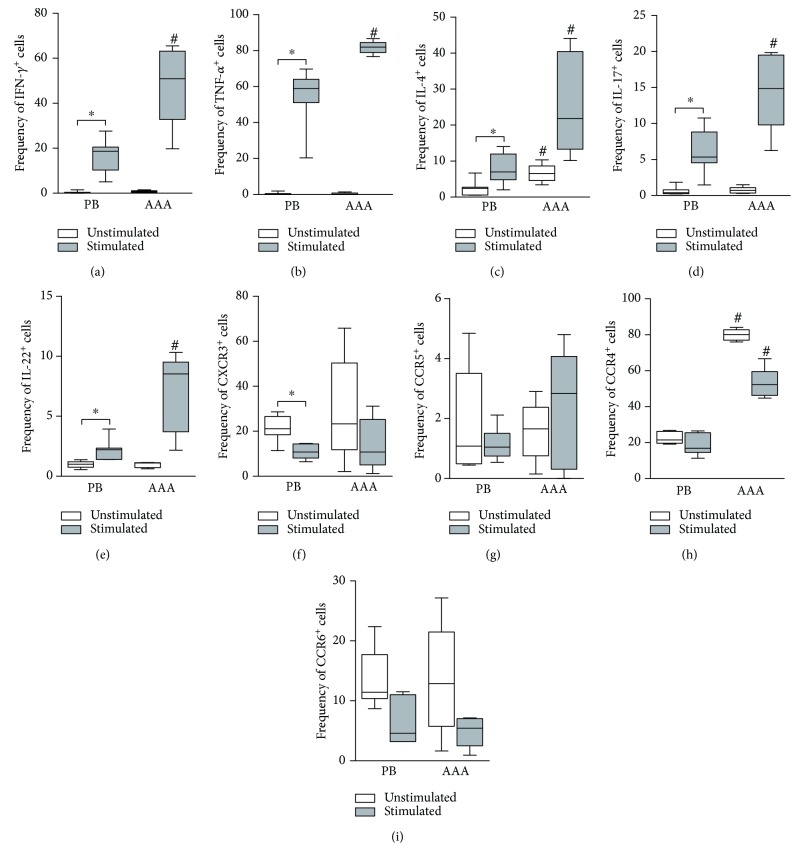
Comparatives analysis of CD4^+^ lymphocyte (T_H_ cell) populations in peripheral blood (PB) (*N* = 12) and lesions (AAA) (*N* = 5) from patients with abdominal aortic aneurysm regarding the frequency (%) of cells positive for the signature cytokines (a–e) and *homing* molecules (f–i) of the different T_H_ subsets (T_H_1, T_H_2, T_H_17, and T_H_22). Peripheral blood mononuclear cell samples were cultured for 48 h in the absence of stimulus (white) or in the presence of anti-CD3, anti-CD28, and anti-CD2 antibody-coated beads, with the addition of PMA plus calcium ionophore in the last 6 h of incubation (grey). The mononuclear cell infiltrates (AAA) were expanded *in vitro* for 15 days and left without stimulation (white) or were stimulated by PMA plus calcium ionophore in the last 6 h of incubation (grey). *P* < 0.05; ^∗^comparison between stimulated and nonstimulated cells (Wilcoxon matched pairs test); ^#^comparison between PB and AAA cells (Mann-Whitney test).

In addition, we compared lesional and peripheral blood T_H_ cells collected from the same AAA patients prior to and after *in vitro* stimulations (Figure 4). In the unstimulated samples, we found an increased percentage of IL-4^+^ T_H_ lymphocytes within lesions compared to peripheral blood (Figure 4(c)). The results were similar for CCR4, which was more frequent in T_H_ cells from lesions (Figure 4(h)). After activation, increased frequencies of T_H_ cells with inflammatory potential were detected in lesions. In fact, we found higher percentages of IFN-*γ* ^+^TNF-*α* ^+^ T_H_1, IL-4^+^ T_H_2, IL-17^+^ T_H_17, and IL-22^+^ T_H_22 cells in situ than in peripheral blood (Figures 4(a)–4(e)). The chemokine receptor CCR4 was also more frequent in the lesional T_H_ population (Figure 4(h)).

## 4. Discussion

In the present study, we examined lesions from patients with AAA to get a reliable scenario regarding disease pathogenesis. To achieve this aim, we enrolled only patients subjected to open surgical repair of aneurysms. The criteria for the procedure largely explain the clinical features of our experimental groups: aneurysm repair must be performed when the damaged aorta is either more than 5.5 cm wide or grows more than 8 mm a year. Additionally, only patients without major cardiovascular, respiratory, or renal comorbidities are enrolled for the open surgical technique [31]. In agreement with these prerequisites, the average diameter of the aorta in our experimental group was 6.5 cm (advanced stage lesions), and the patients did not show serious relevant comorbidities. Moreover, biochemical parameters were all within the normal range, except for C-reactive protein. The patterns observed regarding age, sex, and comorbidities were expected based on prior epidemiological studies [32].

We detected T_H_1, T_H_2, T_H_17, and T_H_22 cells in mononuclear cell infiltrates extracted from AAA lesions using flow cytometry. Although in low numbers, IFN-*γ* ^+^TNF-*α* ^+^ T_H_1, IL-4^+^ T_H_2, IL-17^+^ T_H_17, and IL-22^+^ T_H_22 cells were detected in unstimulated samples. After *in vitro* stimulation, we found a high number of T_H_ cells producing their signature cytokines. The most prevalent subset was T_H_1 (IFN-*γ* ^+^TNF-*α* ^+^), followed by T_H_2 (IL-4^+^), T_H_17 (IL-17^+^), and T_H_22 (IL-22^+^). The absence of a significant number of IL-17^+^IL-22^+^ cells suggests that IL-22 production is carried out by T_H_22 cells in AAA tissue. It has been shown that these different subsets migrate to lesions through different receptors: CXCR3/CCR5 (T_H_1) and CCR4/CCR6 (T_H_2, T_H_17, and T_H_22) [16, 33, 34]. In our study, we found that CXCR3^+^ cells were more prevalent than CCR5^+^ or CCR5^+^CXCR3^+^ cells in unstimulated and stimulated T_H_ populations from AAA lesions, suggesting that T_H_1 cells may migrate mainly via CXCR3. In fact, the most frequent receptor in the T_H_ cells was CCR4. We did not detect a significant percentage of CCR4^+^CCR6^+^ cells, which prevented further analysis of the pathophysiology of the T_H_17 and T_H_22 subsets, because these subtypes are double positives for these receptors. The high prevalence of CCR4^+^ T_H_ cells in mononuclear infiltrates from AAA lesions suggests that the main receptor for T_H_2 migration is CCR4, and this subset appears to be the most frequent one in AAA lesions. Collectively, the results regarding AAA lesions show the presence of all T_H_ subsets in situ.

Similar to the results of other studies, by IHC staining, we found CD3^+^CD8^−^ T cells mainly in follicle-like structures localized in the media and adventitia of AAA tissue sections [6, 35]. Indeed, we observed positive staining for either IL-4, IL-17, IL-23, or IL-22 colocalizing with CD3^+^CD8^−^ T cells. Thus, we were able to confirm the presence of different activated T cell subsets within AAA lesions. Interestingly, we also detected IL-23 in macrophage-like cells in the tunica intima. In accordance, a previous paper by Sharma et al. showed elevated IL-17 and IL-23 expressions in aortic tissue of AAA patients compared with controls, as measured by PCR and ELISA [36].

Several reports have associated T_H_ subtypes with the pathophysiology of AAA [35, 37]. These studies focused mainly on the T_H_1 and T_H_2 populations and showed that their signature cytokines (IFN-*γ* and IL-4, respectively) were able to induce the production and activation of different sets of enzymes involved in extracellular matrix degradation, such as MMPs and cathepsins [35, 38]. Interestingly, many of these studies were based on animal model experiments and present conflicting data. Xiong and colleagues found that IFN-*γ* knockout mice had smaller lesions compared to wild-type animals as a consequence of lower MMP production by macrophages because of the lack of IFN-*γ* [39]. However, another study using murine aortic transplantation showed that wild-type allograft segment hosts specifically elicited an IFN-*γ*-predominant response within the engraftment but failed to develop AAA. Conversely, transplantation into hosts lacking the IFN-*γ* receptor led to an IL-4-dominated response with medial elastin loss and AAA development. The authors concluded that IFN-*γ* plays a beneficial role in the context of AAA, because it regulates the deleterious IL-4-driven response [40].

More recent studies attempted to show the role of T_H_17 cytokine IL-17 in AAA development; however, the results are contradictory. Romain et al. used a model for SOCS3 overexpression in T cells and showed that diminished IL-17 production promoted severe AAA in mice [41]. Contrarily, the inhibition of IL-17 by digoxin, in a model of porcine pancreatic elastase perfusion in C57BL/6 mice, promoted protection against AAA [42]. Such findings are probably a result of the application of different animal models, combined with the use of inflammatory agents such as elastase, angiotensin II, or calcium chloride infusion, for studying AAA pathogenesis and thus may not adequately reflect human AAA development [38].

We also analyzed the different T_H_ subsets in the peripheral blood from patients and from control subjects by flow cytometry. We found a low number of T_H_ cells positive for their signature cytokines in both groups. However, after *in vitro* stimulation, we found that the percentages of IFN-*γ* ^+^TNF-*α* ^+^ (T_H_1), IL-4^+^ (T_H_2), IL-17^+^ (T_H_17), and IL-22^+^ (T_H_22) cells increased. The results were similar for control subjects, except for IL-4^+^ (T_H_2), whose percentage did not increase. However, we noticed that AAA patients had higher percentages of all T_H_ subsets capable of producing their signature inflammatory cytokines compared to the control participants. Thus, our results show for the first time that an increased inflammatory potential of peripheral blood T_H_ cell populations in AAA patients may contribute to the systemic low-grade inflammatory process observed as the high serum CRP levels in AAA patients.

Regarding the chemokine receptors, our results showed that the most frequent receptors in peripheral blood T_H_ cells from patients with AAA were CXCR3 and CCR4. Most of the cells were single positive, and the frequency of all receptors decreased after stimulation. In peripheral blood from control subjects, we found a high percentage of CXCR3^+^CCR5^+^ cells and CCR4^+^ cells, while CCR6 was the less frequent receptor. Unlike in AAA patients, the prevalence of all receptors increased in peripheral blood T_H_ cells after stimulation. It is interesting to note that AAA patients had higher percentages of unstimulated cells that are positive for CXCR3 and CCR6 and that the frequency of CCR5^+^ cells is nearly inexistent in the absence of stimulation. Collectively, these results may reflect differences between AAA patients and control subjects regarding prior activation of peripheral blood. In the control group, the majority of cells may be naive, while cells from AAA patients may be mainly preactivated memory cells.

We also compared lesional and peripheral blood cells from same patients in order to associate peripheral and in situ immune responses. Regarding the unstimulated cells, we found no differences between lesional and peripheral blood samples, except for IL-4^+^ and CCR4^+^ cells (T_H_2). After *in vitro* stimulation, lesional samples had increased frequencies of the different T_H_ subsets expressing their signature cytokines compared to peripheral blood samples. In addition, CCR4 was more prevalent in stimulated lesional samples. These results suggest that T_H_ subsets are preferentially activated in lesions and that the inflammatory process is mainly local.

## 5. Conclusions

Overall, our results clearly show the presence of the classic T_H_ subsets (T_H_1 and T_H_2) and of the recently discovered T_H_ subsets (T_H_17 and T_H_22) in AAA lesions. Furthermore, we demonstrated that the presence of an increased amount of peripheral blood T_H_ cells capable of producing their proinflammatory signature cytokines might contribute to the low-grade inflammatory process occurring in patients with AAA. However, we suggest that the activation of the different T_H_ subsets preferentially takes place within AAA lesions.

## Figures and Tables

**Table 1:** Demographic and clinical features of patients with AAA and controls.

	Patients (*N* = 30)	Controls (*N* = 11)	*P*
Age (mean ± SD)	63.4 ± 13.2	60.6 ± 9	0.04
Sex (M/F)	28/2	6/5	0.009
Maximum aortic diameter (mean ± SD)	6.5 ± 1.6	—	—
Maximum thrombus thickness (mean ± SD)	2.8 ± 2.0	—	—
Comorbidities (%)			
Diabetes	23.3	63.6	0.06
Dyslipidemia	20	63.6	0.07
Hypertension	70	81.8	1.0
Smoking history	80	54.6	0.69
Obesity	20	63.6	0.01
Medication (%)			
*β*-Blockers	33.3	36.4	0.17
Aspirin	25	27.3	1.0
ACEI	45.8	36.4	0.68
Calcium blockers	12.5	18.2	0.5
Statins	25	72.8	0.001
Diuretics	20.8	36.4	0.06
Hypoglycemics	20.8	27.3	0.08
Other	33.3	36.4	1.0
Cardiovascular disease (%)	36.0	9.0	0.22
Biochemical parameters (mean ± SD)			
ALT (U/l)	15.3 ± 9.6	31 ± 12.2	0.04
AST (U/l)	19.7 ± 4.3	21.4 ± 4.6	0.39
CR (mg/dl)	1.1 ± 0.3	0.85 ± 0.1	0.03
K (mEq/l)	4.7 ± 0.4	4.8 ± 0.4	0.75
Na (mEq/l)	141.4 ± 3.0	141.3 ± 4.2	0.96
U (mg/dl)	36.1 ± 9.7	32.8 ± 10.2	0.5
CRP (mg/l)	4.68 ± 3.3	2.49 ± 1.8	0.09

Continuous data are presented as mean (standard deviation) and analyzed by *t*-test. Fisher's exact test was used for the analyses of categorical variables. M: male; F: female; SD: standard deviation; ACEI: angiotensin-converting enzyme inhibitors; CAD: coronary artery disease; ALT: alanine aminotransferase; AST: aspartate aminotransferase; CR: creatinine; K: potassium; Na: sodium; U: urea; CRP: C-reactive protein.

## Data Availability

The datasets generated and/or analyzed during the current study are available from the corresponding author on reasonable request.

## Supplementary Materials

Supplementary Materials Details on the primary antibodies used in immunohistochemistry are available in Supplementary Table 1. Details on the antibodies used for extracellular and intracellular stainings (flow cytometry) are available in Supplementary Table 2.
