# Supplementary material for: Characterization of CD4+ T Cell Subsets in Patients with Abdominal Aortic Aneurysms
Source: Mediators Inflamm. 2018 Dec 27;2018:6967310. doi: 10.1155/2018/6967310 (PMC6327259; doi:10.1155/2018/6967310)
Supplement: Supplementary Materials — Details on the primary antibodies used in immunohistochemistry are available in Supplementary Table 1. Details on the antibodies used for extracellular and intracellular stainings (flow cytometry) are available in Supplementary Table 2. [file 6967310.f1.pdf]

Supplementary Table 1. Antibodies for Immunohistochemistry

Protein name	Antibody catalogue number	Vendor	Species	Usage Concentration
CD3	A0452	Dako	Rabbit polyclonal	1.2 µg/mL
CD8	M7103	Dako	Mouse IgG1 monoclonal	2 µg/mL
IL-17	sc7927	Santa Cruz	Rabbit polyclonal	0.8 µg/mL
IL-22	ab134035	Abcam	Mouse IgG1 monoclonal	5 µg/mL
IL-4	500702	Biolegend	Mouse IgG1 monoclonal	5 µg/mL
IL-23	511202	Biolegend	Mouse IgG1 monoclonal	2.5 µg/mL

Supplmentary Table 2. Antibodies for flow cytometry

Protein name	Fluorochrome	Antibody catalogue number	Vendor	Species	Usage volume for [1x10^7cells/mL]
CD3	FITC	MHCD0301	Caltag	Mouse IgG2a monoclonal	1 µL
CD3	PE	MHCD0304	Caltag	Mouse IgG2a monoclonal	1 µL
CD3	PerCP	300326	Biolegend	Mouse IgG2a monoclonal	1 µL
CD8	FITC	555634	BD Bioscience	Mouse IgG1 monoclonal	4 µL
CD8	APC	300912	Biolegend	Mouse IgG1 monoclonal	2 µL
CCR5	FITC	555992	BD Bioscience	Mouse IgG2a monoclonal	4 µL
CCR6	PE	353410	Biolegend	Mouse IgG2b monoclonal	1 µL
CXCR3	PE	557185	BD Bioscience	Mouse IgG1 monoclonal	4 µL
CCR4	PerCP/Cy5.5	359406	Biolegend	Mouse IgG1 monoclonal	1 µL
IFN-γ	FITC	300449	BD Bioscience	Mouse IgG2b monoclonal	2 µL
TNF-α	PE	559321	BD Bioscience	Mouse IgG1 monoclonal	4 µL
IL-4	APC	500714	Biolegend	Mouse IgG1 monoclonal	2 µL
IL-17	PE	12717942	e-Bioscience	Mouse IgG1 monoclonal	1.5 µL
IL-22	eFluor660	50722942	e-Bioscience	Mouse IgG1 monoclonal	1 µL
